# Excessive matrix metalloprotease-mediated degradation of interstitial tissue (type I collagen) independently predicts short-term survival in an observational study of postmenopausal women diagnosed with cancer

**DOI:** 10.18632/oncotarget.15275

**Published:** 2017-02-11

**Authors:** Nicholas Willumsen, Cecilie L. Bager, Stephanie N. Kehlet, Katrine Dragsbaek, Jesper S. Neergaard, Henrik B. Hansen, Anne-Christine Bay-Jensen, Diana J. Leeming, Allan Lipton, Claus Christiansen, Morten Karsdal

**Affiliations:** ^1^ Nordic Bioscience A/S, Biomarkers & Research, Herlev, Denmark; ^2^ Department of Endocrinology, University of Southern Denmark, Odense M, Denmark; ^3^ Division of Hematology/Oncology, Penn State Hershey Medical Center, Pennsylvania State University, Hershey, PA, USA

**Keywords:** MMP, type I collagen, ECM, cancer, mortality

## Abstract

Extensive tissue remodeling mediated by matrix metalloproteases (MMPs) is an important part of cancer. The aim of this study was to investigate whether serum biomarkers reflecting MMP-mediated degradation of type I collagen (C1M), type IV collagen (C4M) and citrullinated vimentin (VICM) were predictive of cancer-specific mortality. Between 1999 and 2001, 5855 Danish postmenopausal women participated in The Prospective Epidemiologic Risk Factor (PERF I) study. Demographics and serum samples were collected at enrolment. Cancer diagnosis, and cause and time of death were obtained from Danish registries. C1M, C4M and VICM were measured by ELISA. Hazard ratios (HR) and Kaplan-Meier curves were applied to assess mortality at 3 and 12 years of follow-up for women diagnosed with cancer within 3 years from blood sampling. Within 3 years from blood sampling, 250 women had been diagnosed with cancer. C1M and VICM were associated with survival over time at 3 years of follow-up. Only C1M was predictive of mortality at 3 years follow-up: the adjusted HR was 2.65 [95% CI: 1.08-6.51]. In conclusion, C1M and VICM are associated with survival in postmenopausal women with cancer, and C1M is an independent risk factor for cancer-specific mortality. Thus, quantification of tissue remodeling is important in cancer.

## INTRODUCTION

A major contributor to mortality in the elderly population is age-related diseases such as cancer [1, 2]. It is therefore an important health priority to identify risk factors for cancer related death in individuals suffering from this disease. Biomarkers for risk assessment may contribute directly to the identification of high-risk subgroups of a population and contribute to understanding the etiology of cancer in more detail [3]. Moreover, such risk factor biomarkers may have a direct impact on clinical decision-making and hence have a significant influence on overall outcome.

An important part of the malignant changes in cancer is extensive tissue remodeling. Importantly, the extracellular matrix (ECM) composition changes and becomes uncontrolled [4]. The ECM is the non-cellular component of all tissues and organs and is divided into the basement membrane and the underlying interstitial matrix. The ECM is constantly being modified and remodeled through protein synthesis and secretion, enzymatic degradation, and post-translational modifications [5].

In tumors the ECM interacts with cells and regulates diverse functions associated with malignancy. It is becoming widely accepted that signals from the ECM affects directly the hallmarks of cancer [6]. Hence, any ECM dysregulation may be part of the changes that drive cancer [7].

ECM remodeling in cancer is characterized both by an increased and altered production of ECM proteins (desmoplasia), as well as an increased and altered degradation of ECM proteins in the presence of proteases such as matrix metalloproteases (MMPs) [8]. Our research group has previously developed a technology enabling non-invasive assessment of MMP-mediated type I collagen degradation (C1M) [9] and MMP-mediated type IV collagen degradation (C4M) [10]. Elevated levels of C1M and C4M have been observed in serum from patients with malignant diseases of the lung, breast, ovary and pancreas [11–13]. Type I collagen and type IV collagen are the main components of interstitial matrix and basement membrane, respectively.

Using the same technology as for C1M and C4M, assessment of MMP-degraded and citrullinated vimentin (VICM) [14] has been found elevated in serum from lung cancer patients [12]. Vimentin is an intermediate filament protein. It is released from activated stromal cells [15] and expressed on the surface of cancer cells [16] making it a possible MMP substrate and target for citrullination by peptidylarginine deiminase (PAD) which are activated during inflammation and cancer [17].

Recently C1M was shown to be predictive of mortality in a large observational study of postmenopausal women, the Prospective Epidemiological Risk Factor (PERF I) study [18]. The best predictive value of C1M was for cancer-specific mortality, the most prevalent cause of death. Interestingly, it was also recently shown in the PERF I cohort, that C1M, C4M, and VICM were elevated in those women that were subsequently diagnosed with cancer and that C1M and VICM were both independent predictors of increased risk of developing cancer [19]. It remains to be determined if the predictive value of C1M for cancer-specific mortality also applies to women given a cancer diagnosis within the same vicinity of time, as well as it remains to be determined if C4M and VICM predicts cancer-specific mortality in this cohort. In this study, we sought to determine if C1M, C4M and VICM was predictive of cancer-specific mortality in the subgroup of women in the PERF I study who were diagnosed with cancer shortly after PERF I enrolment, i.e. within 3 years from blood sampling.

## RESULTS

### Cohort definition and baseline characteristics

**Figure 1:**
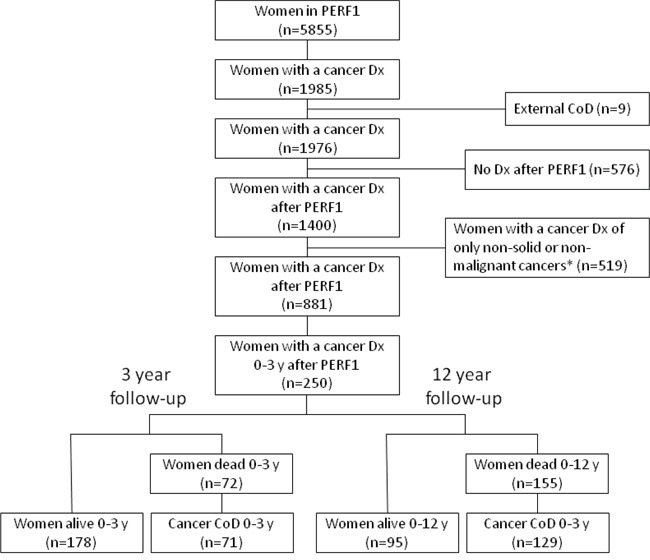
Cohort and study design Overview of women included and excluded from analysis. *Excluded were those diagnosed with a benign neoplasm: ICD10 codes D10-D36; neoplasms of uncertain or unknown behavior: ICD10 codes D37-D48; skin cancer: ICD-10 codes C44, D040-D049 (i.e. not including malignant melanoma); dysplasia: ICD-10 code N87; non-solid tumors: ICD-10 codes C81-C96 (a.k.a. malignant neoplasms stated or presumed to be primary, of lymphoid, hematopoietic and related tissue). External CoD: ICD-10 codes V01-Y98; Cancer CoD: ICD10 codes C00-D48. CoD =cause of death.

The study design and women included and excluded from analysis are summarized in Figure 1. In total, 34% (n=1985) of the women participating in PERF I were diagnosed with cancer at any time between 1947 and 2012, the latest date recorded in the Danish registries. Of the women diagnosed with cancer after PERF I enrollment (n=881), 28% (n=250) were diagnosed within the first 3 years. This cohort of women was used to investigate survival in the first 3 years from blood sampling and in the entire follow-up period of approximately 12 years. Of the 250 women diagnosed in the first 3 years, 29% (n=72) died within the first 3 years. In 99% of these (n=71), cancer was registered as the CoD. During the entire 12-year follow-up, 62% (n=155) died in total with 83% (n=129) having cancer registered as the CoD.

The characteristics of the women diagnosed with cancer within the first 3 years from baseline are summarized in Table 1. The women are stratified as ‘alive’, ‘dead by all-cause mortality’ and ‘dead by cancer-specific mortality. Details are outlined for 3 and 12 years’ follow-up.

**Table 1:** Cohort characteristics of women diagnosed with cancer within the first three years from baseline for 3 and 12 years of follow-up

			*3 year follow-up*					*12 year follow-up*				
Variable		Total n=250	Alive n=178	Dead (All) n=72	*p-value vs. Alive*	Dead (Cancer) n=71	*p-value vs. Alive*	Alive n=95	Dead (All) n=155	*p-value vs. Alive*	Dead (Cancer) n=129	*p-value vs. Alive*
Age at baseline (years)		72.5 [71.3-73.8] (250)	72.4 [71.0-73.8] (178)	73.0 [70.9-75.5] (72)	*0.214*	73.1 [71.1-75.6] (71)	*0.116*	71.3 [69.9-72.5] (95)	73.8 [72.0-75.1] (155)	*0.004*	73.2 [71.3-74.7] (129)	*0.014*
BMI (≥25)		55 % (133/241)	60 % (104/173)	43 % (29/68)	*0.021*	42 % (28/67)	*0.016*	61 % (57/93)	51 % (76/148)	*0.168*	51 % (62/122)	*0.164*
Smoking		27 % (68/250)	24 % (42/178)	36 % (26/72)	*0.063*	37 % (26/71)	*0.054*	21 % (20/95)	31 % (48/155)	*0.118*	29 % (37/129)	*0.254*
Alcohol (≥7 drinks/week)		36 % (89/249)	35 % (62/177)	38 % (27/72)	*0.823*	38 % (27/71)	*0.765*	35 % (33/94)	36 % (56/155)	*0.979*	39 % (50/129)	*0.677*
Exercise (≥1 time/week)		65 % (162/249)	70 % (124/177)	53 % (38/72)	*0.014*	52 % (37/71)	*0.011*	68 % (64/94)	63 % (98/155)	*0.521*	62 % (80/129)	*0.427*
Education:	Primary school High school University	73 % (183/249) 21 % (51/249) 6 % (15/249)	75 % (132/177) 18 % (33/177) 7 % (12/177)	71 % (51/72) 25 % (18/72) 4 % (3/72)	*0.430*	70 % (50/71) 25 % (18/71) 5 % (3/71)	*0.411*	72 % (68/94) 18 % (17/94) 10 % (9/94)	74 % (115/155) 22 % (34/155) 4 % (6/155)	*0.164*	72 % (93/129) 24 % (31/129) 4 % (5/129)	*0.157*
Hypertension		6 % (15/250)	7 % (12/178)	4 % (3/72)	*0.630*	4 % (3/71)	*0.647*	5 % (5/95)	6 % (10/155)	*0.913*	7 % (9/129)	*0.807*
Hyperlipidemia		35 % (88/249)	39 % (69/178)	27 % (19/71)	*0.101*	26 % (18/70)	*0.073*	40 % (38/95)	32 % (50/154)	*0.184*	30 % (39/128)	*0.181*
HRT		23 % (57/250)	24 % (42/178)	21 % (15/72)	*0.760*	21 % (15/71)	*0.801*	26 % (25/95)	21 % (32/155)	*0.378*	20 % (26/129)	*0.355*
C1M (ng/ml)		42.9 [39.1-47.5] (238)	40.9 [38.2-45.8] (171)	47.3 [38.4-54.8] (67)	*0.080*	46.8 [38.4-53.3] (66)	*0.092*	42.9 [38.8-50.5] (91)	42.3 [38.1-47.8] (147)	*0.561*	43.8 [38.3-48.9] (121)	*0.971*
C4M (ng/ml)		71.9 [68.6-75.8] (238)	73.2 [68.7-76.2] (171)	69.2 [66.4-78.6] (67)	*0.975*	69.1 [66.4-78.6] (66)	*0.806*	75.3 [70.7-79.2] (91)	71.0 [66.3-75.6] (147)	*0.127*	69.0 [66.1-76.9] (121)	*0.160*
VICM (ng/ml)		3.9 [3.60-4.30] (238)	3.60 [3.12-4.00] (171)	4.70 [3.90-6.10] (67)	*0.002*	4.75 [3.96-6.14] (66)	*0.002*	3.80 [3.37-4.10] (91)	3.90 [3.43-4.70] (147)	*0.578*	4.00 [3.62-4.80] (121)	*0.463*

Data are shown as either median with 95% CI in brackets or as percentage. Absolute numbers shown in parentheses (cases/total)

### Overall characteristics of the cancer diagnoses in the cohort

The most prevalent cancers were malignant neoplasms of the digestive organs (ICD-10 codes C15-C26), the respiratory and intra-thoracic organs (ICD-10 codes C30-C39), the breast (ICD-10 codes C50) and the female genital organs (ICD-10 codes C51-C58), which together accounted for 76% of all diagnoses within the first 3 years from baseline. In the 250 women diagnosed within the first 3 years, a total of 253 diagnoses were registered. Of these, 115 were defined as local disease, 76 as lymph node-positive, 42 as metastatic and 19 as unknown. Median biomarker levels did not differ significantly between localized cancers and those in later stages (data not shown). More localized cancers and less metastatic cancer were found in the women still alive than in those who died. Significantly fewer days from baseline to diagnosis were observed in the deceased group compared with those still alive at 12 years follow-up whereas no difference was detected at 3 years follow-up. Finally, of the 250 women diagnosed within the first 3 years, 16% (n=41) had a cancer diagnose registered prior to PERF I.

### Variables as predictors of cancer-specific mortality within 3 years and 12 years from baseline

The individual ability of C1M, C4M, VICM and common risk factors to predict cancer-specific mortality at 3 years and 12 years of follow-up, are shown in Table 2 which summarizes HRs with 95% CIs calculated from univariable Cox proportional-hazard regression models. At 3 years follow-up, C1M and VICM, but not C4M, were predictive of cancer-specific mortality when dividing the biomarkers into quartiles. BMI, smoking and exercise were the only secondary risk factors associated with cancer-specific mortality at 3 years follow-up. At 12 years follow-up, none of the 3 biomarkers were associated with cancer specific mortality; only age and smoking were associated with cancer-specific mortality at 12 years follow-up.

**Table 2:** Individual association between biomarkers and common risk factors for cancer, and cancer-specific mortality after 3 and 12 years of follow-up for women diagnosed within the first 3 years from baseline

Variable		*3-year follow-up*				*12-year follow-up*			
		n (dead/total)	HR	95%CI	*p-value*	n (dead/total)	HR	95%CI	*p-value*
C1M	continuous 21.2-32.1 ng/ml, Q1 32.2-42.8 ng/ml, Q2 42.9-63.1 ng/ml, Q3 63.2-372.5 ng/ml, Q4	66/237	1.01 1.00 2.37 2.23 2.49	1.00-1.01 - 1.08-5.18 1.01-4.94 1.13-5.48	*0.060* - *0.032* *0.049* *0.024*	121/212	1.00 1.00 1.35 1.31 1.18	0.99-1.01 - 0.81-2.26 0.78-2.20 0.70-2.00	0.397 - 0.252 0.313 0.543
C4M	continuous 26.6-60.0 ng/ml, Q1 60.1-71.8 ng/ml, Q2 71.9-90.0 ng/ml, Q3 90.1-210.6 ng/ml, Q4	66/237	1.00 1.00 1.45 0.79 1.11	0.99-1.01 - 0.76-2.77 0.38-1.64 0.56-2.22	*0.722* - *0.263* *0.537* *0.761*	121/212	1.00 1.00 1.12 0.84 0.76	0.99-1.00 - 0.69-1.82 0.51-1.39 0.46-1.27	0.383 - 0.639 0.504 0.301
VICM	continuous 0.7-2.2 ng/ml, Q1 2.3-3.9 ng/ml, Q2 4.0-6.5 ng/ml, Q3 6.6-26.2 ng/ml, Q4	66/237	1.01 1.00 1.54 2.25 3.10	0.99-1.02 - 0.68-3.51 1.06-4.80 1.48-6.49	*0.454* - *0.302* *0.036* *0.003*	121/212	1.00 1.00 0.90 1.05 1.37	0.99-1.02 - 0.54-1.52 0.64-1.73 0.85-2.21	0.361 - 0.705 0.839 0.195
Age at baseline		71/249	1.03	0.99-1.07	*0.112*	129/224	1.04	1.01-1.07	0.007
BMI (≥25)		67/240	0.53	0.33-0.86	*0.010*	122/215	0.67	0.48-0.98	0.038
Smoking (yes/no)		71/249	1.76	1.09-2.85	*0.022*	129/224	1.48	1.01-2.16	0.045
Alcohol (≥7 drinks/week)		71/248	1.13	0.70-1.82	*0.620*	129/223	1.04	0.71-1.51	0.860
Exercise (vs ≥1 time/week)		71/248	0.52	0.33-0.82	*0.006*	129/223	0.75	0.53-1.07	0.113
Education:	Primary school (Ref.) High schoolUniversity	71/248	1.00 1.37 0.67	- 0.78-2.29 0.21-2.13	- *0.286* *0.496*	129/223	1.00 1.24 0.53	- 0.82-1.85 0.22-1.31	- 0.307 0.171
Hypertension (yes/no)		71/249	0.61	0.19-1.94	*0.409*	129/224	1.01	0.51-1.98	0.975
Hyperlipidemia (yes/no)		70/248	0.59	0.35-1.01	*0.053*	128/223	0.72	0.50-1.05	0.091
HRT (yes/no)		71/249	0.90	0.51-1.59	*0.716*	129/224	0.82	0.53-1.26	0.362
A cancer Dx prior to PERF1 (yes/no)		71/249	1.45	0.82-2.55	*0.204*	129/224	1.65	1.08-2.50	0.020

Hazard ratios (HR) were calculated by univariable analysis. Biomarkers were analyzed on both a continuous scale and divided into quartiles with the lower quartile (Q1) used as a reference to calculate the HR for women in the three upper quartiles (Q2-Q4). All risk factors, except for age, were analyzed on a binominal scale. BMI = body mass index; HRT = hormone replacement therapy; Dx = diagnosis.

**Figure 2:**
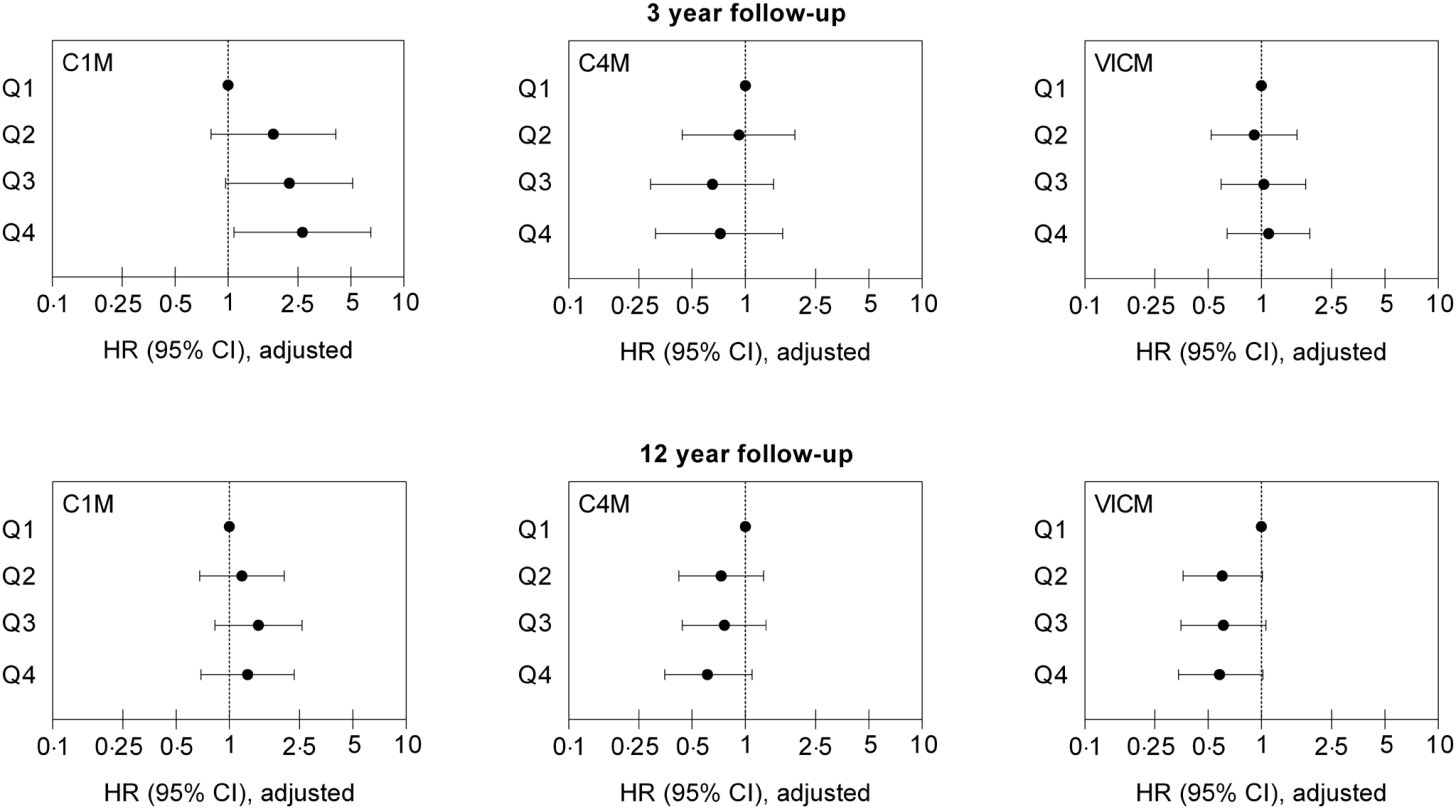
Independent predictive value of C1M, C4M and VICM for cancer-specific mortality at 3 and 12 years follow-up Hazard ratios (HR) with 95% confidence intervals (95% CI) for cancer-specific mortality in quartiles (Q1–Q4) of C1M, C4M and VICM are shown. Values are adjusted for age, BMI, smoking status, alcohol consumption, exercise level, educational level, hypertension, hyperlipidemia, use of HRT, and history of cancer.

To address the potential independent predictive value of C1M, C4M and VICM for cancer-specific mortality, a multivariable Cox proportional-hazard regression model was used to calculate HR for the biomarkers divided into quartiles and including the secondary risk factors listed in Table 2. The HRs with 95% CIs are illustrated in Figure 2, and details of all variables in the model are summarized in Table 3. At 3 years’ follow-up, only C1M was independently associated with cancer-specific mortality. The risk was stepwise increased with increasing quartile and women having the highest C1M levels (Q4) had a 2.65-fold (HR=2.65) higher risk of cancer-specific mortality than the women with the lowest C1M levels (Q1). In contrast, neither VICM nor C4M was independently associated with cancer-specific mortality. At 12 years’ follow-up none of the three biomarkers were independently associated with cancer-specific mortality. Moreover, none of the three markers were independently associated with cancer-specific mortality when analyzed on a continuous scale (data not shown).

**Table 3:** Independent association between biomarkers and common risk factors for cancer, and cancer-specific mortality after 3 and 12 years of follow-up for women diagnosed within the first 3 years from baseline

Variable		*3-year follow-up*				*12-year follow-up*			
		n (dead/total)	HR	95%CI	*p-value*	n (dead/total)	HR	95%CI	*p-value*
C1M	21.2-32.1 ng/ml, Q1 32.2-42.8 ng/ml, Q2 42.9-63.1 ng/ml, Q3 63.2-372.5 ng/ml, Q4	63/230	1.00 1.81 2.23 2.65	- 0.80-4.10 0.97-5.13 1.08-6.51	- *0.153* *0.061* *0.035*	117/205	1.00 1.18 1.46 1.27	- 0.68-2.05 0.83-2.58 0.69-2.33	- 0.564 0.193 0.440
C4M	26.6-60.0 ng/ml, Q1 60.1-71.8 ng/ml, Q2 71.9-90.0 ng/ml, Q3 90.1-210.6 ng/ml, Q4		1.00 0.92 0.65 0.72	- 0.44-1.91 0.29-1.44 0.31-1.63	- *0.816* *0.289* *0.426*		1.00 0.73 0.76 0.61	- 0.42-1.27 0.44-1.31 0.35-1.09	- *0.269* *0.327* *0.095*
VICM	0.7-2.2 ng/ml, Q1 2.3-3.9 ng/ml, Q2 4.0-6.5 ng/ml, Q3 6.6-26.2 ng/ml, Q4		1.00 0.91 1.03 1.10	- 0.52-1.59 0.59-1.78 0.64-1.88	- *0.736* *0.928* *0.741*		1.00 0.60 0.61 0.58	- 0.36-1.01 0.35-1.06 0.34-1.02	- *0.053* *0.078* *0.060*
Age at baseline			1.02	0.98-1.06	*0.331*		1.04	1.01-1.08	0.018
BMI (≥25)			0.56	0.33-0.95	*0.032*		0.69	0.47-1.01	0.058
Smoking (yes/no)			1.61	0.91-2.84	*0.105*		1.31	0.84-2.06	0.228
Alcohol (≥7 drinks/week)			1.00	0.58-1.72	*0.999*		1.00	0.67-1.47	0.994
Exercise (vs ≥1 time/week)			0.54	0.30-0.91	*0.021*		0.79	0.53-1.19	0.269
Education:	Primary school (Ref.) High schoolUniversity		1.00 1.24 0.89	- 0.65-2.38 0.26-3.05	- *0.515* *0.853*		1.00 1.11 0.62	- 0.69-1.80 0.24-1.59	- *0.660* *0.319*
Hypertension (yes/no)			0.60	0.17-2.04	*0.412*		0.80	0.37-1.78	0.601
Hyperlipidemia (yes/no)			0.51	0.28-0.94	*0.032*		0.66	0.43-1.01	0.058
HRT (yes/no)			0.83	0.44-1.55	*0.349*		0.74	0.46-1.19	0.223
A cancer Dx prior to PERF1 (yes/no)			1.12	0.58-2.16	*0.730*		1.51	0.94-2.43	0.088

Hazard ratios (HR) were calculated by multivariate analysis. Biomarkers were analyzed on both a continuous scale and divided into quartiles with the lower quartile (Q1) used as a reference to calculate the HR for women in the three upper quartiles (Q2-Q4). All risk factors except for age were analyzed on a binominal scale. BMI = body mass index; HRT = hormone replacement therapy; Dx = diagnosis.

### Association between C1M, C4M and VICM levels, and survival over time

**Figure 3:**
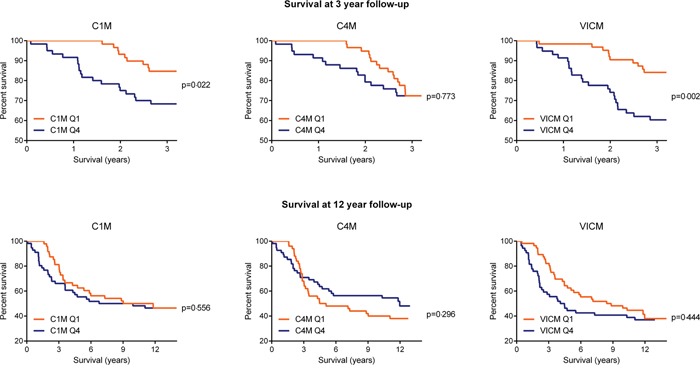
Percentage of women surviving cancer at 3 and 12 years follow-up, by C1M, C4M and VICM biomarker levels at baseline Kaplan-Meier survival curves illustrated the percent survival over time for women with baseline biomarker levels in the upper quartile (Q4) vs the lower quartile (Q1). A log-rank test was used to determine differences between the survival curves. Curves considered significantly different if p<0.05.

Kaplan-Meier survival curves illustrate cancer-specific mortality over time for the three biomarkers analyzed (Figure 3). Only women with biomarker levels in Q1 and Q4 were compared. At 3 years’ follow-up, a significant difference was detected in the survival curves for C1M (p=0.022) and VICM (p=0.002) but not for C4M (p=0.773). No significant difference was detected for the entire follow-up period.

## DISCUSSION

In this study we investigated whether the serum-based biomarkers of MMP-degraded type I collagen (C1M), MMP-degraded type IV collagen (C4M) and MMP-degraded citrullinated vimentin (VICM) were associated with cancer-specific mortality in a cohort of postmenopausal women diagnosed with cancer within 3 years from blood sampling. For both C1M and VICM, the women with the highest biomarker levels (Q4) had a significantly lower survival rate within the first 3 years of follow-up as compared to women having the lowest biomarker levels (Q1). However, only C1M, but not VICM, was able to independently predict cancer-specific mortality in this cohort. The predictability for C1M only applied to the outcome 0-3 years after blood sampling, and not 0-12 years after blood sampling, indicating that C1M could reflect ongoing progressive cancer-related activity.

C1M is the sum of increased type I collagen production and degradation and could be regarded as a marker of more active ECM or interstitial matrix remodeling. C1M could be regarded as a potential surrogate measure of a specific desmoplastic event and increased proteolytic (MMP) activity degrading type I collagen. Moreover, type I collagen have been shown to be degraded by MMPs as part of cell invasion and morphogenesis *in vitro* [20]. It is also well established that interstitial collagenases (MMPs) are associated with a poor prognosis in a variety of cancers [21].

The lack of ability to detect an association between C4M and cancer-specific mortality may seem counterintuitive, as basement membrane degradation, producing C4M is typically associated with tumor/epithelial cell invasion and metastasis, which again is associated with a poorer outcome than localized tumors. It is possible that other basement membrane degradation products will be associated with cancer progression and mortality in the present study.

The fact that relatively high C1M levels prior to diagnosis are associated with mortality indicates that interstitial matrix damage may lead to a more tumor-progressive stromal environment. Interestingly, different studies indicate that the underlying stroma, which includes the interstitial matrix, rather than the epithelium, is the target of carcinogens. In one example, a malignant transformation of epithelial cells occurred only when the stroma was exposed *in vivo* to a carcinogen [22]. In another example, when epithelial cells were transplanted into irradiated BALB/c mice, tumors occurred more quickly and grew larger than in non-irradiated animals [23]. These findings suggest that cancer is more than just a result of epithelial cell proliferation and breaching of the basement membrane (invasion). It is a dynamic and adaptive phenomenon taking place at the tissue level and highlights the importance of addressing the composition and quality of the stroma including the ECM. An emerging concept suggests that an altered tissue architecture is in fact the core of carcinogenesis [24].

As type I collagen is found in many tissues, and as C1M is a circulating fragment of it, it remains to be established whether C1M is derived solely from local tumor tissue remodeling or from a more systemic effect on many tissues and organs. C1M has been found elevated in several different diseases characterized by ECM remodeling and inflammation and consequently it is not specific for cancer [25–31]. This, together with the fact that this cohort included women not presenting with cancer at time of blood sampling, could indicate that high levels of C1M are indicative of an more general unhealthy phenotype, that is, with excessive interstitial matrix remodeling. The present findings could enable one to argue that this unhealthy phenotype could be an underlying and under-recognized mechanism in cancer leading to a poor prognosis and decreased survival following a cancer diagnosis. In support of this, a novel paradigm suggests that cancer originates following a sequence of events that include chronic inflammation and desmoplasia with associated changes in the cellular microenvironment [32]. The present data, together with the previously published PERF I data showing that C1M, C4M, and VICM were associated with increased risk of developing cancer [19], supports this notion of a role of chronic inflammation and desmoplasia as part of tumorgenesis. Thus, ECM and tissue remodeling may be associated with a pre-disposition of being diagnosed with a cancer later in life, and consequently affecting the survival, as the tumor microenvironment is more permissive of cancer development and progression. A better outcome for cancer patients might be achieved by reversing the underlying mechanisms that result in high levels of ECM turnover and tissue remodeling. In fact, in a study of rheumatoid arthritis [27], a treatment-induced decrease in C1M levels by >35 % was associated with a lack of disease progression. Whether similar findings will be seen in cancer patients remain to be established.

Other serum biomarkers of ECM turnover and tissue remodeling have been linked with mortality. Although not focusing solely on women diagnosed with cancer, high serum levels of endostatin, a fragment of type XVIII collagen, have been found to be associated with increased mortality risk in two independent cohorts of elderly people with a follow-up of 8 and 9 years, respectively [33]. Likewise, although not focusing on elderly people, elevated plasma levels of YKL-40, a protein that is thought to play a role in inflammation and tissue remodeling, have been shown to predict increased mortality risk during 14 years of follow-up of women diagnosed with cancer [34].

The current study had some limitations. The cohort consisted of elderly Danish postmenopausal women, and it is yet to be investigated whether these results would be seen in other ages, gender and ethnic groups. Women who volunteer for epidemiological studies may over-represent the proportion of relatively healthy subjects in the general population and may bias the findings, albeit in favor of rejecting our hypothesis. As some of the baseline characteristics (e.g. smoking habits) were collected from self-reported questionnaires this may introduce a response/recall bias, however this is minimized by using current status of the patients (e.g. current smoking status). In the current study, multiple cancer types with unknown medical history were included. It is above the scope of the present study to investigate individual cancer types in relation to mortality. Still, it is important to recognize that the apparent prognostic effect of C1M may be influenced by cancer type, specific treatment regimens and history of intervention. The contribution of co-morbidities remains to be determined and although C1M seems to have prognostic value, the true clinical utility of C1M remains to be established.

In conclusion, we have shown that C1M and VICM are associated with survival over time in elderly women who are diagnosed with cancer up to 3 years after blood sampling. Moreover, C1M is an independent risk factor for cancer-specific mortality. Women with high baseline levels of C1M who were diagnosed with cancer within 3 years of blood sampling had approximately 3-fold increased risk of death within the first 3 years of follow-up compared with those with low baseline C1M levels. This supports the potential of C1M and VICM as biomarkers of a poor short-term outcome in cancer and underlines the importance of measuring altered ECM and tissue remodeling to understand the development and progression of this disease.

## MATERIALS AND METHODS

### Study design and participants

Between 1999 and 2001, a total of 5855 postmenopausal Danish women aged 60-85 participated in the PERF I, an observational, prospective cohort study aiming to identify risk factors associated with age-related diseases [18]. All subjects were derived from a database (n=8875) of postmenopausal women previously invited to participate in clinical trials at the Center for Clinical and Basic Research (CCBR) in Copenhagen or Aalborg. To ensure that no overrepresentation of women with a history of any specific diseases occurred, all women in the CCBR database were invited irrespective of previous medical history. A total of 5855 women volunteered to participate in PERF I. The study was approved by the local ethics committees and carried out in accordance with ICH-GCP and the Helsinki declaration. Informed consents were obtained from all individual participants.

### Baseline investigations

A fasting venous blood sample was drawn at the PERF I baseline visit. A self-reported questionnaire was used with standardized methods to capture demographic and other baseline data including age, body mass index (BMI), current smoking habits, socioeconomic status, medical history, physical activity level, current alcohol intake, and level of education.

### Cancer diagnoses

Cancer diagnoses were collected from the Danish Cancer Registry in December 2014 in which the last diagnosis was registered in December 2012. Diagnoses were classified according to the International Statistical Classification of Diseases (ICD). Diagnoses were based on ICD10 between 1977 and 2012, and ICD7 for the period prior to 1977. Only women diagnosed with cancer 0-3 years after baseline were included in the analyses. Women with only benign neoplasms (D10-D36), neoplasms of uncertain or unknown behavior (D37-D48), non-melanoma skin cancer (C44, D040-D049), dysplasia (N87), malignant neoplasms stated or presumed to be primary, of lymphoid, hematopoietic and related tissue (C81-C96), and any successive combination of only those diagnoses were excluded from the group of women diagnosed with cancer.

### Serum biomarkers analysis

A central CAP-certified laboratory measured levels of MMP-degraded type I collagen (C1M), MMP-degraded type IV collagen (C4M) and MMP-degraded citrullianted vimentin (VICM) in serum using competitive enzyme-linked immune sorbent assays (ELISAs). Assays measuring C1M, C4M and VICM, which each have a specific MMP-generated neoepitope present, were originally described and validated by Leeming et al [9], Sand et al [10], and Vassiliadis et al [14], respectively. Technically, the intra- and inter-assay variations are <10 % and <15 % for these assays. Levels of biomarkers were determined in duplicates in samples stored for approximately 12 years at −80°C. Women missing biomarker measurements were excluded from the analysis.

### Primary end-point (outcome variable)

Cancer-specific mortality (using ICD10 codes C00-D48) was the primary end-point. Data on cause and time of death up to 1 January 2013 were collected from the National Danish Causes of Death Registry and The Danish Civil Registration System, respectively. Causes of death (CoD) were classified according to ICD10. Deaths due to any causes other than cancer (ICD10 codes D50-U85) were excluded from the risk analysis. Total survival time was defined as years from enrollment in PERF 1 until date of death or being alive at 1 January 2013. The average follow-up was 12.1 years.

### Risk factors (other variables)

The primary risk factors were serum C1M, C4M and VICM levels. Secondary risk factors assessed at baseline included age, BMI (≥25), smoking (current, yes/no), alcohol consumption (current, ≥7 drinks/week), exercise (≥1 time/week), education (primary school, high school, university), hypertension (current, yes/no), hyperlipidemia (current, yes/no), use of hormone replacement therapy (HRT) (current, yes/no).

### Statistical analysis

Baseline characteristics of deceased women and survivors were compared using either a Mann–Whitney *U* test for numerical values or a Chi-square test for categorical values.

Univariable Cox proportional-hazards regression models were used to calculate hazard ratios (HRs) with 95% confidence intervals (95% CI) for cancer-specific mortality for the primary and secondary risk factors for cancer: C1M, C4M, VICM, age, BMI, smoking status, alcohol intake, exercise, level of education, hypertension, hyperlipidemia, use of HRT. To assess any confounding effects, a multivariable Cox proportional-hazard regression model was used to calculate the independent HR with 95% CI for cancer-specific mortality including the primary and secondary risk factors and also adjusted in cases where cancer was diagnosed before PERF I enrollment. Baseline biomarker levels in the lowest quartile (Q1) were used as a reference to calculate the HRs for women with baseline biomarkers levels in the three upper quartiles (Q2-Q4). Univariable analysis also included HRs for C1M, C4M and VICM on a continuous scale. The time-to-mortality intervals analyzed were 0-3 years and 0-12 years from blood sampling.

Kaplan-Meier survival curves were used to analyze mortality over time in the lowest (Q1) and highest (Q4) quartiles. A log-rank test was used to determine differences between curves.

Statistical analysis was conducted using MedCalc^®^ (v12.3.0). Data were considered statistically different when p<0.05.
